# Measuring and evaluating morphological asymmetry in fish: distinct lateral dimorphism in the jaws of scale-eating cichlids

**DOI:** 10.1002/ece3.849

**Published:** 2013-10-24

**Authors:** Hiroki Hata, Masaki Yasugi, Yuichi Takeuchi, Satoshi Takahashi, Michio Hori

**Affiliations:** 1 Graduate School of Science and Engineering, Ehime University2-5 Bunkyo, Matsuyama, Ehime, 790-8577, Japan; 2 Center for Ecological Research, Kyoto University509-3, 2-chome, Hirano, Otsu, Shiga, 520-2113, Japan; 3 Graduate School of Medicine and Pharmaceutical Sciences, University of Toyama2630 Sugitani, Toyama, 930-0194, Japan; 4 Research Group of Information and Computer Sciences, Division of Natural Sciences, Nara Women‘s UniversityKitauoya Nishimachi, Nara, 630-8506, Japan; 5 Graduate School of Science, Kyoto UniversityKitashirakawa-Oiwake, Sakyo, Kyoto, 606-8502, Japan

**Keywords:** Morphological laterality, *Perissodus microlepis*, scale-eater, Tanganyikan cichlid

## Abstract

The left–right asymmetry of scale-eating Tanganyikan cichlids is described as a unilateral topographical shift of the quadratomandibular joints. This morphological laterality has a genetic basis and has therefore been used as a model for studying negative frequency-dependent selection and the resulting oscillation in frequencies of two genotypes, lefty and righty, in a population. This study aims were to confirm this laterality in *Perissodus microlepis* Boulenger and *P. straeleni* (Poll) and evaluate an appropriate method for measuring and testing the asymmetry. Left–right differences in the height of the mandible posterior ends (HMPE) and the angle between the neurocranium and vertebrae of *P. microlepis* and *P. straeleni* were measured on skeletal specimens. Snout-bending angle was also measured using a dorsal image of the same individuals following a previous method. To define which distribution model, fluctuating asymmetry (FA), directional asymmetry (DA), or antisymmetry (AS), best fit to the lateral asymmetry of the traits, we provided an R package, IASD. As a result, HMPE and neurocranium–vertebrae angle of both species were best fitted to AS, suggesting that *P. microlepis* and *P. straeleni* showed a distinct dimorphism in these traits, although snout-bending angle of *P. microlepis* was best fitted to FA. Measurement error was low for HMPE comparing the snout-bending angle in *P. microlepis*, indicating that measuring HMPE is a more accurate method. The scale-eating tribe Perissodini showed distinct antisymmetry in the jaw skeleton and neurocranium–vertebrae angle, and this laterality remains a valid marker for further evolutionary studies.

## Introduction

The bilateral asymmetry of the scale-eating Tanganyikan cichlid *Perissodus eccentricus* was first described as “bilateral differential growth rates of two bones in the jaw suspension and the remodeling of articular surfaces of the lower jaw joints” (Liem and Stewart 1976). The mechanical and integrated asymmetries of the cranial skeleton of this taxon are clearly demonstrated in the figures in Liem and Stewart (1976). The direction of the mouth opening in adult individuals is readily apparent when viewed from the ventral side (see Figure 11 in Liem and Stewart (1976)). Other scale-eating cichlids, *Perissodus microlepis* and *P. straeleni*, are well known because of their morphological asymmetry and associated behavioral handedness in scale-eating. For example, a lefty opens its mouth toward to the right and attacks the left side of prey fishes when attacking from behind (Hori 1993; Takahashi et al. 2007; Takeuchi et al. 2012). This mouth laterality is thought to facilitate efficient scale-eating because of the increased contact area between the predator's teeth and the flank of prey fish. The mouth asymmetry is shared by all seven cichlids within the genus *Perissodus*: *P. microlepis*, *P. straeleni*, *P. hecqui* (Boulenger), *P. multidentatus* (Poll), *P. elaviae* (Poll), *P. paradoxus* (Boulenger), and *P. eccentricus* (Hori 1991, 1993). The frequency of lefty and righty morphs in natural populations of *P. microlepis* was found to fluctuate around an approximately 50:50 ratio over more than a decade of sampling, and negative frequency-dependent selection has been invoked to explain the relative stability of this ratio (Hori 1993). Specifically, the more common morph is thought to suffer a fitness disadvantage because prey fish would be attacked more often from the preferred side by the scale-eaters, making them more alert to attacks originating from that direction. Therefore, the more common morph of the predator would be expected to succeed less often in removing scales than the rare morph, which would in turn be favored (Hori 1993).

Kusche et al. (2012), however, questioned this bimodal morphological laterality in *P. microlepis* based on their findings that the snout-bending angle measured on the dorsal images of this fish was not clearly distinct between lefty and righty morphs, and the frequency distribution of the angle was unimodal distribution. This study tests the hypothesis that morphological asymmetry in *P. microlepis* is clearer in the lower jaw joints and the neurocranium–vertebrae angle than the snout-bending angle. We focused on opening in-lever of lower jaws (Albertson et al. 2005) and the neurocranium–vertebrae angle to assess morphological asymmetry because these two measures are based on mechanically functional structure in the skeletal morphology. We aim to confirm the bilateral asymmetry in *P. microlepis* and a congeneric scale-eater, *P. straeleni* and present an appropriate method for measuring and testing the morphological asymmetry.

## Materials and Methods

### Field sampling of specimens

We sampled 50 adult individuals of *P. microlepis* and 10 adult individuals of *P. straeleni* from Kasenga Point (8º43′S, 31º08′E) near Mpulungu (Zambia) on the southern tip of Lake Tanganyika in November 2012. This is one of the sites where Kusche et al. (2012) collected the same species. Specimens were caught with gill nets. All of the fishes were killed in chilled water, gently boiled and preserved in 10% formalin solution.

### Measurement of bilateral asymmetry

**Figure 1:**
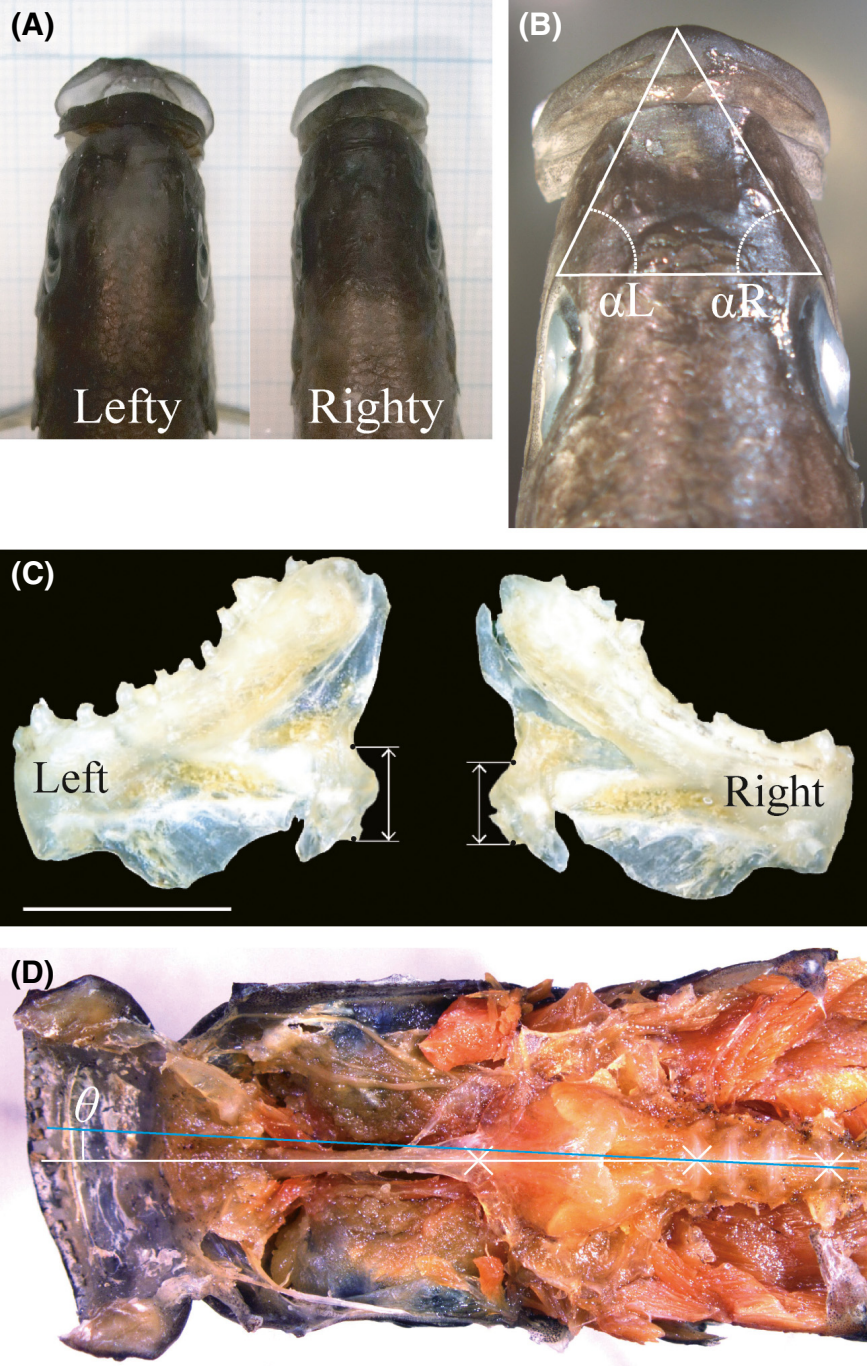
Dorsal view of lefty and righty of *Perissodus microlepis* (A), the triangle connecting the frontal points of the two eye pits and the upper jaw intersection and measured angles of the right (αR) and left (αL) corners (B), the height of the mandible posterior ends (HMPE, C) and the angle from the vertebrae to the neurocranium (*θ*, D).

First, we measured snout-bending angle using the images of the specimens of *P. microlepis* (Fig. 1A and B). A picture of each individual in dorsal view was taken in the laboratory using a digital microscope VHX-100 (Keyence Cooperation, Osaka, Japan). We measured mouth asymmetry following Hori et al. (2007) and Kusche et al. (2012). We drew a triangle connecting the frontal points of the two eye pits and the upper jaw intersection on each fish head image and measured the angles of the right (αR) and left (αL) corners to the nearest 0.1º, after which “αL − αR” was calculated. Two pictures were taken for each specimen, and the measurement was made on both images.

Bilateral asymmetry of the same specimens and specimens of *P. straeleni* was also quantified by two additional measures based on skeletal morphology: the left–right difference in the height of the mandible posterior ends (HMPE, Fig. 1C) and the angle from the vertebrae to the neurocranium (*θ*, Fig. 1D). Tissues were carefully removed by hand to prepare the skeletal material. The datum points were marked with a fine pen on each skeletal specimen under a binocular microscope, and HMPE and *θ* were measured using a digital microscope (VHX-100; Keyence Cooperation) within 0.005 mm and 0.05º of accuracy, respectively. The two datum points were maintained in a horizontal position with the original devices (Figure S1). The specimen was secured with a small clip that had a universal joint connected to a base, the entirety of which was placed on a two-dimensional goniometric stage. It was leveled by manipulating the goniometric stage and by monitoring through a scope level adapted from a microscope. We used the average value of two measurements from each specimen, and each measurement was obtained by repositioning the specimen on the microscope stage. An index of asymmetry (IAS) was calculated as follows: [2 × (*R* − *L*)/(*R* + *L*)] × 100, where *R* and *L* are the heights of the right and left mandibles, respectively (Hori et al. 2007). Individuals with *R > L* were designated as “righty” because their right sides dominated over the left, and the IAS was assigned a positive value. In contrast, individuals with *R* < *L* were designated as “lefty”, with a negative IAS. For the neurocranium–vertebrae angle (*θ*), an individual in which the right side of the head faced front with the neurocranium bent toward the right in ventral view was designated as “righty”, and *θ* was given a positive value. In contrast, an individual in which the left side of the head faced front and the neurocranium bent toward the left in ventral view was designated as “lefty”, and *θ* was given a negative value.

### Estimation of measurement error

Measurement error (ME) caused by photography were analyzed using two indices: ME1 and ME3. ME1 is the average difference between paired values measuring the same trait twice (Palmer and Strobeck 2003). Two people (H. H. and M. Y.) measured the same trait independently. ME1 was calculated using Σ|*M*_1_ − *M*_2_|/*n*, where *M*_1_ and *M*_2_ are the values for the first and the second measurements, respectively, and *n* is the number of samples. ME3 was calculated by (MS_error_/MS_sides × individuals_) × 100 from the two-way mixed-model ANOVA on each trait with sides (left or right) as a fixed factor and individuals as a random factor (Palmer and Strobeck 2003). MS_error_ and MS_sides × individuals_ indicate within-individual mean squares (MS) and sides × individuals interaction MS, respectively, in this ANOVA. MS3 is the percentage of measurement error to nondirectional asymmetry.

ME1 and ME3 were also calculated for HMPE and neurocranium–vertebrae angle (*θ*) on skeletal images and compared with those calculated for the external dorsal images.

### Statistical test for morphological asymmetry

To test the frequency distribution of the three indices of morphological laterality measured in this study, we conducted a model selection method for the distribution patterns (Hata et al. 2011; Yasugi and Hori 2011). The distributions were fitted to the following three models: fluctuating asymmetry (FA), with a normal distribution, mean = 0, and SD of data; directional asymmetry (DA), with a normal distribution, mean ≠ 0, and SD of data; and antisymmetry (AS), with two normal distributions, means (mean_1_ = −mean_2_), and SD (SD_1_ = SD_2_) calculated by the maximum-likelihood estimation. The Akaike information criterion (AIC) was calculated for each model, and the model with the lowest AIC was considered to be the distribution type (FA, DA, or AS) for each distribution pattern. This best-fit model selection for the trait frequency distribution is provided here as an R package IASD_1.0.7.

To compare our results to those of previous studies, we used the same statistical methods as Van Dooren et al. (2010) and Kusche et al. (2012). The unimodality of the distributions were analyzed by dip test (Hartigan and Hartigan 1985) using an R package, diptest_0.75–4. A mixture analysis was conducted to investigate whether trait frequency distributions were composed of one or two component distributions using an R package, mixtools_0.4.6 (McLachlan and Peel 2000; Benaglia et al. 2009). To detect “weak antisymmetry” in which trait frequency distributions have a wide range (Van Valen 1962), the degree of kurtosis was evaluated by an Anscombe–Glynn test (Anscombe and Glynn 1983) using an R package, moments_0.13. The means of indices were analyzed for departure from zero to test for directional asymmetry using a one-sample *t*-test. Using repeated measurements from replicate images that were used for the estimation of ME, the above-mentioned two-way mixed-model ANOVA on each trait with sides (left or right) as a fixed factor and individuals as a random factor was carried out to test if the asymmetry exhibits DA or non-DA. This analysis test for the significance of DA (effects of “sides”) and non-DA (effects of “side × individuals”) relative to the variation of measurement error. All the statistical analyses were conducted using R version 3.0.0 (R Core Team 2013).

## Results

**Figure 2:**
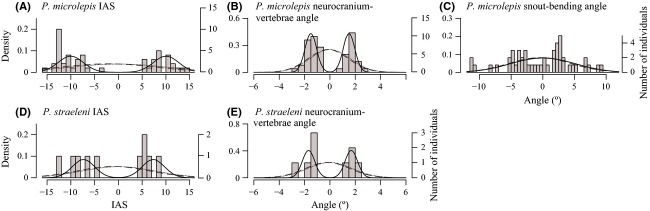
Frequency distribution of mouth asymmetry in *Perissodus microlepis* and *Perissodus straeleni*. The lines quantified by the left *y*-axis show the probability curves derived from the three models: the FA model (dotted line), DA model (broken line), and AS model (solid line).

Jaw asymmetry and the angle between the neurocranium and vertebrae of *P. microlepis* and *P. straeleni* showed discrete dimorphism (Fig. 2A, B, D, and E). The bimodal antisymmetry model was selected as the best model for both IAS of the HMPE and neurocranium–vertebrae angle in both species, but not for snout-bending angle in *P. microlepis* (Table 1).

**Table 1:** AIC values for the FA, DA, and AS models to discriminate the type of asymmetry for three indices in two cichlid species, *Perissodus microlepis* and *Perissodus straeleni*: IAS of mandibles, neurocranium–vertebrae angle, and snout-bending angle

					AIC for each model		
					
Species	Index	*n*	Mean	SD	FA	DA	AS
Pmic	IAS	50	10.0	2.7	377.8	377.4	**313.2**
Pmic	Neurocranium–vertebrae angle	50	1.5	0.5	189.4	191.0	**137.9**
Pmic	Snout-bending angle	49	4.0	2.8	**296.7**	298.2	298.6
Pstr	IAS	10	7.3	2.5	71.2	73.0	**63.3**
Pstr	Neurocranium–vertebrae angle	9	1.7	0.5	37.7	39.6	**29.2**

Bold indicates the minimum value among the AICs for the three models. Pmic, *Perissodus microlepis*; Pstr, *Perissodus straeleni*.

The statistical results revealed antisymmetry in the IAS of the mandibles and the neurocranium–vertebrae angle, but not in the snout-bending angle for *P. microlepis* (Table 2). The trait mean was always not significantly different from zero, suggesting no DA in all the indices of both species. The dip test found that for the IAS of the mandibles and the neurocranium–vertebrae angle in *P. microlepis* trait frequency distributions were significantly different from a unimodal distribution, but that the snout-bending angle showed a unimodal distribution. The Anscombe–Glynn tests revealed significant platykurtosis for the IAS and the neurocranium–vertebrae angle of *P. microlepis*, but not for the snout-bending angle. Mixture analysis also suggested that the mixture of two normal distributions was the best fit for the frequency distribution of the IAS and the neurocranium–vertebrae angle in *P. microlepis*, but that a single-component distribution was the best fit for the snout-bending angle. In contrast, for *P. straeleni*, the dip test detected significance only for the neurocranium–vertebrae angle, and the Anscombe–Glynn tests found significance only for the IAS. Mixture analysis suggests unimodal distributions were the best fit for both indices for *P. straeleni*. The two-way mixed-model ANOVA confirmed that DA was not valid for all the traits of both *P. microlepis* and *P. straeleni* (Table 3).

**Table 2:** Statistical analyses of laterality indices related to body asymmetry in *Perissodus microlepis* and *Perissodus straeleni*. Analyses include the dip tests for unimodality, one-sample *t-*tests for the assessment of the deviation of the trait mean from zero, Anscombe–Glynn test for platykurtosis and mixture analyses to determine the number of components

Index	*n*	Trait mean	Dip test		One-sample *t*-test		Platykurtosis		Mixture analysis	
			
			Dip	*P*-value	*t*-test	*P*-value	Kurtosis	*P*-value	*n* Components	*P*-value
(a) *Perissodus microlepis*										
IAS	50	−2.236	0.115	<0.001	−1.546	0.129	1.314	<0.001	2	0.869
Neurocranium–vertebrae angle	50	−0.140	0.122	<0.001	−0.623	0.536	1.431	<0.001	2	0.504
Snout-bending angle	49	−0.476	0.064	0.124	−0.677	0.502	2.685	0.874	1	0.976
(b) *Perissodus straeleni*										
IAS	10	−1.063	0.133	0.084	−0.418	0.686	1.304	0.023	1	0.064
Neurocranium–vertebrae angle	9	−0.189	0.176	0.004	−0.306	0.767	1.376	0.060	1	0.065

**Table 3:** Statistical tests for the significance of DA (“side”) and non-DA (“side × individual”; FA and AS) relative to measurement error (residuals) using two-way mixed-model ANOVA (side = fixed, individual = random factor)

Effect	DF	SS	MS	*F*	*P-*value
(a) *Perissodus microlepis*_IAS					
Side	1	0.114	0.114	3.173	0.081
Individual	49	13.801	0.282	259.006	<0.001
Side × individual	49	1.754	0.036	32.916	<0.001
Residuals	100	0.109	0.001		
(b) *Perissodus microlepis* snout-bending angle					
Side	1	11.086	11.086	0.458	0.502
Individual	48	508.966	10.603	3.047	<0.001
pSide × individual	48	1162.170	24.212	6.957	<0.001
Residuals	98	341.079	3.480		
(c) *Perissodus straeleni*_IAS					
Side	1	0.003	0.003	0.151	0.707
Individual	9	0.406	0.045	446.942	<0.001
Side × individual	9	0.193	0.021	212.059	<0.001
Residuals	20	0.002	0.000		

For *P. microlepis*, the ME1 of angles αL and αR was 1.9 and 2.6, respectively. ME3 of the angles, the average difference between replicate measurements as a percent of the average difference between sides was 14.4% (Table 3), similar to the result in a previous study on the same fish species (Kusche et al. 2012). The ME1 of HPME of left jaws and right jaws were 0.019 and 0.010, respectively, and the ME3 was 3.1%. For *P. straeleni*, the ME1 of HPME of left and right jaws was 0.016 and 0.007, respectively, and the ME3 was 0.5%.

## Discussion

Our measurements of skeletal morphology (i.e., HMPE in the lower jaws and neurocranium–vertebrae angle) clearly showed distinct dimorphism in a population of *P. microlepis*, although the snout-bending angle showed a unimodal distribution indicating FA. Measurement error was low for the HMPE comparing αL and αR in both species, indicating that the HMPE was more accurate index for measuring the morphological laterality in these fishes. Our model selection for the frequency distribution of IAS in HMPE and neurocranium–vertebrae angle confirms distinct lateral dimorphism in the two scale-eating Tanganyikan cichlids, *P. microlepis* and *P. straeleni*. Note, however, that some statistics indicated no significant difference from FA in these indices for *P. straeleni* probably due to the limited number of samples.

A discrepancy is apparent between the measurement of snout external shape and that of skeletal shapes. The earliest studies established that morphological laterality is most apparent in the lower jaws. *Perissodus* fishes have a thick lip containing a hypertrophied collagenous tract (see Figures 8 and 9 in Liem and Stewart (1976)) to stabilize the abducted jaws. Fixed conditions such as water content of the specimen and/or solute concentration may cause osmotically induced deformation of these soft tissues. Indeed, Kusche et al. (2012) found 11 individuals that showed unusual asymmetry (snout-bending angle > 15º) and excluded them from their analysis because they judged that these specimens had been deformed during preservation. As our previous measurement of the dorsal view of the snout was problematic, we suggest a better measure of morphological laterality based on the skeletal structure of the lower jaws and the joint angle between the neurocranium and vertebrae (Takeuchi et al. 2010, 2012; Hata et al. 2011; Yasugi and Hori 2011). Van Dooren et al. (2010) also analyzed morphological asymmetry in *P. microlepis* using dorsal images of the fish similar to Kusche et al. (2012). They used the posterior edge of the eye socket and the anterior end of the dorsal fin as landmarks, resulting in the detection of a weak antisymmetry in this fish. In addition, Van Dooren et al. (2010) conducted a handedness reformation experiment in which *P. microlepis* in an aquarium were fed with a bilaterally biased dummy prey, a “soft-bait dummy fish wrapped in trout skin and with spikes preventing foraging from the forbidden flank”. They observed one individual change its morphological laterality during the course of the experiment, but the remaining seven individuals showed no change. Based on this experiment, Palmer (2010) supported the hypothesis that morphological laterality in nature is induced by developmental plasticity (Palmer 2004, 2009; Lee et al. 2012) and that laterality in *P. microlepis* is not genetic trait but is induced by handed behavior. However, measurements of skeletal morphology reconfirmed an obvious bimodal asymmetry in the scale-eaters. Asymmetry in the skeletal morphology of *Perissodus* fishes is a mechanical and integrated asymmetry constructed through bilateral developmental differences as described by Liem and Stewart (1976). It would be difficult for this skeletal asymmetry to change during the 6 months of the constraint experiment. In addition, the genetic basis of this morphological laterality has been indicated by examining parents and their offspring in wild species (a goby (Seki et al. 2000) and two cichlids (Hori et al. 2007)) and in breeding experiments (a cichlid (Hata et al. 2012), medaka (Hata et al. 2012) and zebrafish (Hata and Hori 2012)) in which righty and lefty offspring were born in a Mendelian ratio with lefty dominant over righty. Recent molecular investigations have also suggested genomic loci corresponding to mandibular asymmetry (Stewart and Albertson 2010).

Here, we propose that measurements of skeletal morphology in the lower jaws and/or the joint angle between the neurocranium and vertebrae are needed to discriminate the morphological laterality of focal species. The morphological laterality of scale-eating cichlids in Lake Tanganyika shows a clear bimodal distribution. As this laterality has a genetic basis, this obvious dimorphism in a population will provide a marker for further studies on evolution.

## Supporting Information

**Figure S1.** Picture of our devise to measure the HMPE and the angle from vertebrae to the neurocranium.
