# Supplementary figures and images for: Measuring and evaluating morphological asymmetry in fish: distinct lateral dimorphism in the jaws of scale-eating cichlids
Source: Ecol Evol. 2013 Oct 24;3(14):4641–7. doi: 10.1002/ece3.849 (PMC3867900; doi:10.1002/ece3.849)

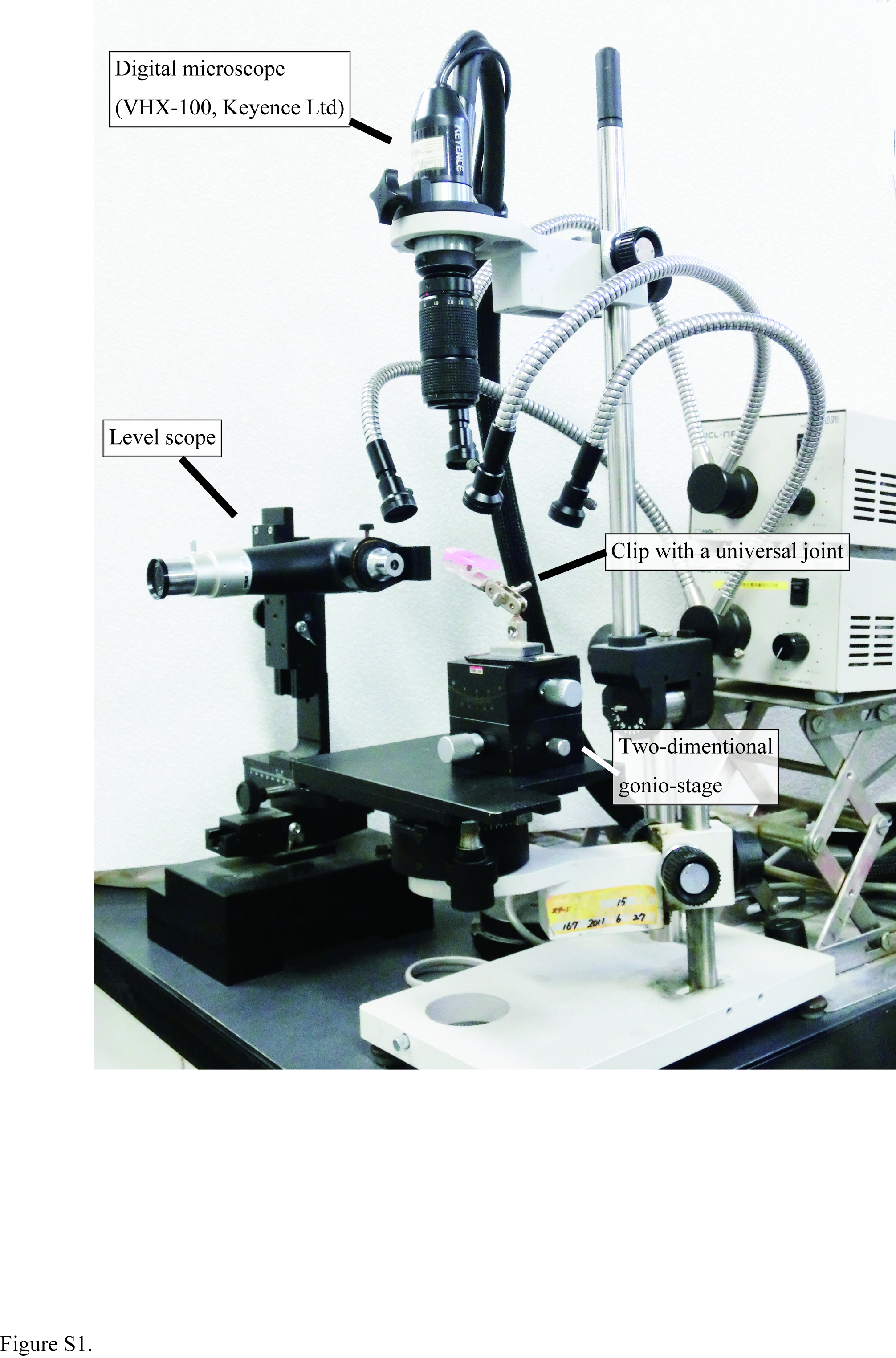

Supplement: Supplementary file 1 [file ece30003-4641-SD1.tif]
